# Quinsy as a Rheumatism

**Published:** 1886-03

**Authors:** J. Suydam Knox

**Affiliations:** Adjunct Professor of Obstetrics and Diseases of Children in Rush Medical College; 14 Loomis street


QUINSY AS A RHEUMATISM. *By* J. SUYDAM KNOX, A. M., M. D.,

*Adjunct Professor of Obstetrics and Diseases of Children in Rush Medical College.*

[Read before the Chicago Medical Society, on February 1st, 1886.]

Quinsy, an acute tonsilitis, is an inflammation of the parenchyma of one or both tonsils, with their mucous coverings, glandular apparatus, and surrounding connective-tissue. It generally ends in suppuration.

Its immediate cause is invariably exposure to wet or cold.

The prominent symptoms are: a sudden onset, a high temperature (102° to 104° F.), great soreness and stiffness of the parts inflamed, exquisite tenderness on motion (such as swallowing), and great swelling and œdema of the contiguous parts.

Niemeyer, Ziemssen, Aitkins, Bosworth, Flint, Loomis, Bristow, and Bartholow all state that there is a hereditary predisposition to quinsey, and a tendency in those affected to recurrent attacks. Morell Mackenzie and E. Fletcher Ingals, in addition, state that rheumatic individuals are most liable to the disease.

The treatment most approved of by these writers may be thus summed up:

Arterial sedatives, as aconite, veratrum viride and antimony; saline cathartics; warm fomentations externally; and astringent gargles, usually with chlorate of potassa.

Empirically, quinine and cimicifuga internally and guiacum and sodium bicarbonate locally have been used with varied success.

Treatment, however, thus far has been a dismal failure. Trousseau in his inimitable way, thus expresses it:

" Inflammatory sore-throat is one of the diseases which are at once the glory and reproach of all kinds of medical treatment—the reproach, because medicine never prevails against them; and the glory, because they terminate in spontaneous recovery, whatever we do."

I hope in the following pages to prove that acute tonsilitis, in a vast majority of cases, is most likely a rheumatic inflammation, and that, so considered, its treatment is simple, efficient and brilliant.

Four years ago I was called to see a gentleman sick with acute articular rheumatism. While rendering him professional services, his mother, who in the capacity of nurse had been much exposed, was suddenly attacked with quinsy. Without apparent benefit I gave her a saline cathartic. Also, hourly, minim doses of tincture of aconite root, and ten-grain doses of sulphate of quinine every six hours.

Forty-eight hours after the commencement of her illness inflammatory rheumatism seized her. The medicine was changed to eight-grain doses of salicylate of soda hourly. To our mutual surprise, the quinsy disappeared like magic. I was much impressed at the time by the apparent relationship of quinsy and rheumatism, and the prompt relief of the former through anti-rheumatic remedies. Later inquiries developed the facts that a second son was subject to both quinsy and rheumatism, and a daughter to repeated attacks of quinsy, and a son of this daughter to rheumatism.

Since then I have treated forty-nine cases of acute tonsilitis, —making fifty in all.

In each case careful inquiry was made as to rheumatic history, and in 45 cases, or 90 per centum, rheumatism was hereditary in the family. Five cases (ten per centum) had no rheumatic history whatever.

All fifty cases were treated at first with salicylate of soda and aconite, and hot alkaline gargles or the insufflation of bicarbonate of soda.

Forty cases (80 per centum), all rheumatic, recovered under this treatment in from 36 to 72 hours, without suppuration. In some, the effect of the medication was almost immediate. In all, the soreness and exquisite tenderness on swallowing disappeared before the lowered temperature and diminished swelling warranted a cure.

In five cases, positively rheumatic, no benefit accrued from treatment, and all ended in suppuration in from five to eighteen days.

In five cases, positively not rheumatic, quinine was substituted for the salicylate of soda, on account of its failure to relieve. No benefit accrued, and four of the five terminated in suppuration.

Admitting the impropriety of basing definite conclusions upon so small a number of cases, the results are still too startling and positive to be due either to chance or bias, and surely merit further investigation.

Considering for a moment the etiology and symptoms of quinsy, one is impressed by the close resemblance to rheumatism.

The heredity; the proneness to repeated attacks; the immediate cause in exposure to wet and cold; the sudden onset; the high temperature, out of all proportion to the amount of inflammation; the stiffness, and exquisite tenderness on motion, —surely these are familiar features of the graver disease.

Reviewing now the cases briefly reported, one cannot fail to notice the close association of acute tonsilitis with rheumatism. Forty-five out of the fifty, or ninety per centum, possessed a rheumatic diathesis. Since Morell Mackenzie and E. Fletcher Ingals, gentlemen of large experience in throat-affections, call attention to the same fact, it may fairly be assumed that this percentage would be maintained in a larger aggregation of cases.

I take it, that the only logical inference to be drawn is, that the diathesis creates the susceptibility to, and shapes the course of, the disease. Grant this, and quinsy becomes a rheumatic inflammation, and should be treated accordingly.

A second prominent fact in the report of cases is the prompt amelioration of symptoms under anti-rheumatic treatment. This result was so unvarying as to force the conclusion that it was accomplished solely by the remedies used.

Several patients, made wise by experience, commenced using the salicylate of soda and hot gargles as soon as an attack of quinsy threatened, and with unvarying success in aborting the illness. Still further proving the remedial value of anti-rheumatic treatment.

Negative testimony in the same direction is found in the failure of the treatment of the five cases not rheumatic. There are undoubtedly cases of acute tonsilitis which are not due to diathesis, and which specific treatment will not benefit. Such apparently are the five cases reported. The failure of anti-rheumatic medication with these five, brings out more vividly its efficacy with the forty-five.

Were I to formulate anything based on this paper it would be as follows:

1. Acute tonsilitis is generally a rheumatic inflammation.

2. Eighty per centum of such cases can be promptly cured with anti-rheumatic remedies.

3. Acute tonsilitis, not rheumatic, is not yet amenable to treatment.

The following selected cases will illustrate the above propositions:

CASE 1.—Mr. D., retail butcher, aged thirty. For three years he has had attacks of quinsy about every three months. Has had two attacks of acute articular rheumatism, and is of rheumatic family. I was called to see him in March, 1884. He had then been suffering from quinsy nearly twenty-four hours. The tonsils were both immensely swollen, deglutition was very painful, and there was constant expectoration of glairy, ropy mucus from the throat. I administered one minim tr. aconite root and eight grains salicylate of soda each hour, and with an insufflator applied bicarbonate of soda freely to the tonsils. Insufflation was to be practiced each hour, and the soda to be gargled off, a few minutes after, with hot water. Relief was not experienced until evening, about twelve hours, when he ate a fair supper. In forty-eight hours he was at work. He told me it was the first time he had escaped a ten days' illness with suppuration of the tonsils.

CASE 2.—Mrs. W., aged forty-two. Subject to rheumatic neuralgia. Her grandfather and her father died from rheumatic sequelæ. Her only brother now an invalid from valvular heart-disease and rheumatic gout. First attack of quinsy in February, 1883. She was treated by another practitioner. Suppuration and natural cure in five days. Second attack, January 2nd, 1885. Treatment—insufflation of sodii bicarb., hot gargles and hourly doses of sodium salicylate. Relief was immediate. I was notified that a second visit was unnecessary. Third attack in April, 1885. She aborted the disease with the same remedies, without my services.

CASE 3.—Mrs. P., aged twenty-nine. Subject to rheumatism and quinsy for years; whole maternal family rheumatic. Attacked with quinsy January 1st, 1884. I saw her in twenty-four hours. Administered hourly eight-grain doses of salicylate of soda. On account of her inability to open her mouth, hot carbolized lime-water gargles were used instead of insufflation of soda. Recovery occurred in thirty-six hours, without suppuration. She has aborted repeated attacks since then by the same treatment.

Such prompt results, as in these three cases, can only be due to specific treatment.

CASE 4.—Maud P., aged twenty-two. Father's family rheumatic, but she never had rheumatism herself. She has been subject to quinsy for years. Has usually had three or four seizures annually. She was treated by me for quinsy in April, 1884, with salicylate of soda and hot alkaline gargles. Relief came in three days, without suppuration. Since then she has had no attack; each threat of the disease being successfully met by the above remedies.

This result is significant. No attack of quinsy has occurred in nearly two years, as a result of specific treatment, whereas there had previously been a seizure about every three months.

CASE 5.—Nellie M., aged thirty-five. Herself, one sister and father subject to rheumatism. In July, 1883, I was called to treat her for quinsy. Anti-rheumatic treatment as detailed above was faithfully tried for eleven days, when suppuration brought relief. The only effect of the treatment, apparently, was to prolong the suffering and retard suppuration.

CASE 6.—Edw. S., aged twenty-four. No rheumatic history whatever. I was called to treat him for acute double tonsilitis, in September, 1885. Anti-rheumatic treatment was used for thirty-six hours with no benefit, and with much distress from ringing in his ears, and nausea. I substituted quinine, ten grains, and bromide of sodium, 40 grains, in milk, each six hours. Also a gargle of lime-water, carbolic acid (1 per centum) and morphia. Two days following, hot poultices to neck. The acute symptoms were alleviated, but the disease steadily progressed and terminated in suppuration on the eighth day.

14 Loomis street.

